# Homeless on Main Street: Using Photovoice to Highlight Older Adults Among the Hidden Homeless

**DOI:** 10.1093/geroni/igaa057.2583

**Published:** 2020-12-16

**Authors:** H Shellae Versey

**Affiliations:** Wesleyan University, Middletown, Connecticut, United States

## Abstract

Homelessness is a reality for a growing number of Americans living in small towns and rural areas. However, unlike in cities, housing instability may be less visible. Using a photo-elicitation method (i.e., Photovoice), this study explores the meaning of place and obscured visibility to currently and formerly homeless older adults living in a small town in central Connecticut.

Participants (N = 27) were recruited from a local service agency, given cameras and asked to photograph areas around town that were meaningful to them. Photographs were developed and followed by in-person, semi-structured interviews with participants in which photos and experiences during the project were discussed. Primary themes included belonging, generativity, social isolation, and place-making as meaning-making. The study culminated in a community photography exhibition in which photographs from the project were displayed in public spaces around town. Implications for community-based interventions to reach homeless groups in rural areas are discussed. Part of a symposium sponsored by the Qualitative Research Interest Group.

